# Total cardiovascular risk for next 10 years among rural population of Nepal using WHO/ISH risk prediction chart

**DOI:** 10.1186/s13104-017-2436-9

**Published:** 2017-03-07

**Authors:** Mahesh Kumar Khanal, M. S. A. Mansur Ahmed, Mohammad Moniruzzaman, Palash Chandra Banik, Raja Ram Dhungana, Pratiksha Bhandari, Surya Devkota, Arun Shayami

**Affiliations:** 1 Bangladesh Institute of Health Sciences (BIHS), Dhaka, Bangladesh; 2 Department of Community Medicine, Bangladesh Institute of Health Sciences, Dhaka, Bangladesh; 3 Padmakanya Multiple Campus, Tribhuvan University (TU), Kathmandu, Nepal; 4 Institute of Medicine (IOM), Tribhuvan University, Kathmandu, Nepal

**Keywords:** Cardiovascular diseases, WHO/ISH chart, Risk prediction, Total 10-year CVD risk, Rural population

## Abstract

**Background:**

Cardiovascular diseases (CVD) are the leading cause of morbidity and mortality globally. Primary prevention of CVD based on total CVD risk approach using WHO/ISH risk prediction chart would be more effective to stratify population under different risk levels, prioritize and utilize the scarce resources of low and middle-income countries. This study estimated total 10-year CVD risk and determined the proportion of population who need immediate drug therapy among the rural population of Nepal.

**Methods:**

A community based cross-sectional study conducted among 345 participants aged 40–80 years in rural villages of Lamjung District of Nepal. They were selected randomly from total eighteen wards. Data were collected using WHO STEPS questionnaires. WHO/ISH risk prediction chart for SEAR D was used to estimate total cardiovascular risk. Chi-square and independent t-test were used to test significance at the level of p < 0.05 in SPSS version 16.0.

**Results:**

Of the total participants, 55.4% were female. The mean age (standard deviation) of the participants was 53.5 ± 10.1 years. According to WHO/ISH chart proportions of low, moderate and high CVD risk were 86.4%, 9.3%, and 4.3%, respectively. Eleven percent of participants were in need of immediate pharmacotherapy. Age (p = 0.001), level of education (p = 0.01) and occupation (p = 0.001) were significantly associated with elevated CVD risk.

**Conclusion:**

A large proportion of Nepalese rural population is at moderate and high CVD risk. Immediate pharmacological interventions are warranted for at least one in every ten individuals along with lifestyle interventions. Both population-wise and high-risk approaches are required to minimize CVD burden in the future.

## Background

Cardiovascular diseases (CVD) are the major public health problem globally with disproportionately higher occurrence and consequences in developing countries. Data of 2012 showed estimated deaths due to CVD was 17.5 million (31% of total death) with 37% of premature deaths below the age of 70, globally. Almost 80% of such death occurred in low and middle-income countries (LMIC) with the high disparity of premature deaths among such LMIC [1]. In another way, it has been projected that 23.3 million people would die by 2030 only due to CVD [2]. Even in South–East Asia Region (SEAR), CVD accounted for a quarter (25%) of all deaths due to Noncommunicable Diseases (NCD) [3]. Likewise, in Nepal, the prevalence of CVD was 40% among all NCD in-patients in non-specialty hospitals [4].

Population-wise strategies and high-risk strategies are two important methods for prevention of CVD complementing each other [5]. The high-risk strategy identifies individuals who need immediate and sustained care to prevent the development of CVD [5]. The high-risk strategy can be applied through two distinct approaches, first one is targeting and managing each of the single risk factors like hypertension, hyper-cholesterol separately. Another is accounting the total risk of CVD by measuring the cumulative effect of relative risk of all risk factors [6]. However, later one seems to be accurate for identifying a high-risk individual and can enable to guide limited healthcare resources of LMIC cost-effectively [7–9].

Established tools to estimate the total CVD risk are Framingham, SCORE, QRISK, ASSIGN. These tools were developed considering the specific population and might not be applicable to others [10–15]. However, World Health Organization/International Society of Hypertension (WHO/ISH) risk prediction charts have been developed using information on the population distribution of risk factors from WHO Comparative Risk Assessment study of different sub-regions focusing non western developing countries [16, 17]. The chart estimates the total risk of developing fatal and non-fatal cardiovascular diseases (coronary artery disease and stroke) in following 10 years among those people whose CVD were not manifested clinically, categorizes an individual in different risk groups so that the person can be managed only by lifestyle modification or need drug therapy also [16]. The chart is easy to utilize by all physicians and other health workers at all levels of health care. This tool not only helps to make a clinical decision but also estimates and monitor population distribution of total CVD risk [16, 18]. However, there is paucity of evidence on the use of WHO/ISH risk prediction chart in Nepal. Therefore, this study aimed to estimate total 10-year CVD risk and to determine the proportion of population who need immediate drug therapy among the rural community of Nepal using WHO/ISH chart.

## Methods

### Study design and settings

This was a community-based cross-sectional study conducted at Bhotewodar and Sunderbazar Village Development Committee (VDC) of Lamjung between October and November 2014. These villages are about 200 km west from capital city Kathmandu of Nepal and consist of 14,275 population living in 3,869 households. These are the hilly rural areas where agriculture is the main source of livelihood. The sites are the inhabitants of culturally rich people like Gurung, Tamang, Magar, Newar and Dalit, most of them are socially deprived [19].

All eligible men and women were included as the study population. Eligible criteria were age 40–80 years, without having self-reported angina, myocardial infarction, stroke, and intermittent claudication. Pregnant women, mentally retarded and bed-ridden patients were excluded from the study.

### Sample size and sampling technique

The sample size was calculated by using the formula for estimation of proportion for one sample situation. To detect prevalence of 9.5% of moderate and high CVD risk population, as determined by a study conducted in sub-urban municipalities of Nepal [20], the minimum sample size required was 292 with allowable error of 4% and a design effect of 1.5. Taking into consideration a non-response rate of 15%, we decided to select 343 subjects from all male and female population aged 40–80 years (N = 3712).

A cluster sampling technique was used to recruit the required number of participants for the study. Firstly, four wards were selected out of 18 wards using probability proportion to size (PPS). In each ward, we selected the first household from a randomly selected point at the entry of the main road. Then, every 6th household was taken as the study household. All eligible members of each household were listed and one subject was selected randomly for interview by using Kish Table [21].

### Data collection

Five health professionals who had at least intermediate level of health-related education were trained for one week on study protocol, tools and data collection techniques of the study. Data were collected using standard STEPS instrument version 2.2 [21]. The survey tool encompassed questions related to demography and socio-economy, tobacco and alcohol use, dietary intake, physical activity level, and history of high blood pressure and diabetes. The questionnaires were translated into Nepali language and revised after conducting a pilot survey among 20 participants. Blood pressure was measured using doctor’s aneroid sphygmomanometer and stethoscope with an appropriately sized cuff on the unclothed left arm. First reading was taken after 15 min rest-crossing leg and second and third readings were recorded at 5 min interval. Trained technical person collected venous blood, after conforming overnight 12 h fasting to measure blood sugar and lipid profile. All biochemical measurements were done using semi-automated machine of Erba Mannheim (Germany), chem5 v3 model.

### WHO/ISH risk prediction chart

WHO/ISH chart for SEAR D of WHO epidemiological sub-region [22] was used to estimate the total 10-year risk of CVD of all participants. Age, gender, smoking status, systolic blood pressure, presence or absence of diabetes and total serum cholesterol in millimole/liter (mmol/l) were used to compute the total CVD risk. The chart can stratify an individual into low (<10%), moderate (10% to <20%), high (20% to <30%), and very high (>30%) risk groups [16].

### Definitions of variables

A smoker was the one who has smoked currently and who quit smoking less than one year before the assessment [23]. Systolic blood pressure was mean of last two readings. A respondent with diabetes mellitus was defined as the one who had fasting venous blood sugar level 7 mmol/l or who was taking oral hypoglycemic drug or insulin [23, 24]. Caste was categorized into upper caste (Bramhan and Kshetri), Janajati (Gurung, Tamang, Newar, Magar and Thakali) and Dalit (low caste). For occupation, respondent working in paid governmental and nongovernmental organization was labeled as employed, running own business as self-employed, and student or housemaker or non-paid worker as unemployed. Economic status was categorized as poor (<8498) and medium to high (≥8498) economic status according to family income per person per month in Nepalese Rupees (NR) [25].

### Statistical analysis

Data were compiled and entered in Epidata version 3.1. Duplication and omissions of data were checked and edited before coding and exporting into SPSS V.16.0 for further analysis. Descriptive statistics were used to compute different risk categories with 95% confidence interval (CI) for proportion. Mean was calculated with standard deviation (SD) for continuous variables. Chi-square and independent t-tests were conducted to compare categorical and continuous variables, respectively. All tests were two-tail test and p < 0.05 was considered statistically significant.

### Ethical consideration

Ethical clearance was obtained from ethical review committee of Nepal Health Research Council. Written informed consent or thumb impression (if unable to write) were taken from all participants. Study objectives, data collection procedures, benefits and risks of the study, confidentiality, and anticipated use of the results were explained to all participants in detail before taking consent.

## Results

### Socio-demographic characteristics

Total 345 participants were attended in the study. Mean age of study participants was 53.5 ± 10.1 years. The majority of participants were female (55.4%). Forty percent of respondents were in the lowest age group of 40–49 years. Almost all study participants were married (91.3%). More than half of participants (53.6%) did not have formal education. The majority of participants were janajati (55.4%), unemployed (58%). Almost all participants (91.5%) had poor economic status (Table 1).

**Table 1:** Socio-demographic characteristics of respondents

Characteristics	Male, n (%)	Female, n (%)	Total, n (%)	p value
Age in years				
40–49	65 (42.2)	72 (37.7)	137 (39.7)	0.6
50–59	46 (29.9)	59 (30.9)	105 (30.4)	
60–69	32 (20.8)	39 (20.4)	71 (20.6)	
70–79	11 (7.1)	21 (11)	32 (9.3)	
Mean ± SD^b^	53.3 ± 9.9	53.7 ± 10.3	53.5 ± 10.1	0.68
Marital status^a^				
Married	152 (98.7)	163 (98.3)	315 (91.3)	0.001*
Widow or widower	2 (1.2)	28 (14.6)	30 (8.6)	
Level of education^a^				
No formal education	30 (19.5)	155 (81.2)	185 (53.6)	0.001*
Primary school	49 (31.1)	21 (11)	70 (20.3)	
≥Secondary school	75 (48.7)	15 (7.9)	90 (26.1)	
Caste				
Upper caste	67 (43.5)	66 (34.6)	133 (38.6)	0.03*
Janajati	74 (48.1)	117 (61.3)	191 (55.4)	
Dalit	13 (8.4)	8 (4.2)	21 (6.1)	
Occupation^a^				
Employed	33 (21.4)	7 (3.7)	40 (11.6)	0.001*
Self-employed	54 (35.1)	24 (12.6)	78 (22.6)	
Unemployed	42 (27.3)	158 (82.7)	200 (58)	
Retired	25 (16.2)	2 (1)	27 (7.8)	
Economic status				
Poor	135 (39.2)	179 (52.3)	314 (91.5)	0.05*
Medium and high	19 (12.3)	12 (6.3)	31 (9.0)	

Chi square test was done

* Is significant

^a^Fischer-exact test

^b^Independent t test for mean difference

### Total 10-year CVD risk using WHO/ISH chart

The majority of people had low (<10%) 10-year CVD risk [86.4% (CI 82.8–90)]. Moderate and high risk were 9.3% (CI 6.2–12.4) and 4.3% (CI 2.2–6.4), respectively. More female (10.5%) than male (7.8%) had moderate risk. However, more men (4.5%) than women (4.2%) were at high CVD risk (Table 2). Of total study participants, 6 (1.7%) were at >30% CVD risk (Table 3). As expected, total CVD risk was increasing with age. The highest risk was observed among 70–79 years where almost 44% were at moderate risk and 15.6% were at high risk. While only 0.7% were at moderate and high risk at the age of forties (Table 2).

**Table 2:** Distribution of study population into low, moderate and high cardiovascular risk categories, n (%)

Sex	Age (years)	Risk categories		
		Low risk	Moderate risk	High risk
Male	40–49	64 (98.5)	1 (1.5)	0 (0.0)
	50–59	43 (93.5)	2 (4.3)	1 (2.2)
	60–69	24 (75.0)	4 (12.5)	4 (12.5)
	70–79	4 (36.4)	5 (45.5)	2 (18.2)
	Total	135 (87.7)	12 (7.8)	7 (4.5)
Female	40–49	71 (98.6)	0 (0)	1 (1.4)
	50–59	56 (94.9)	2 (3.4)	1 (1.7)
	60–69	27 (69.2)	9 (23.1)	3 (7.7)
	70–79	9 (42.9)	9 (42.9)	3 (14.3)
	Total	163 (85.3)	20 (10.5)	8 (4.2)
Male and Female	40–49	135 (98.5)	1 (0.7)	1 (0.7)
	50–59	99 (94.3)	4 (3.8)	2 (1.9)
	60–69	51 (71.8)	13 (18.3)	7 (9.9)
	70–79	13 (40.6)	14 (43.8)	5 (15.6)
	Total	298 (86.4)	32 (9.3)	15 (4.3)

**Table 3:** Socio-demographic factors associated with elevated 10-year CVD risk, n (%)

Variables	<10% risk (low)	>10% risk (elevated)	p value
Gender			
Male	135 (87.7)	19 (12.3)	0.53
Female	163 (85.3)	28 (14.7)	
Age			
40–49	135 (98.5)	2 (1.5)	0.001*
50–59	99 (94.3)	6 (5.7)	
60–69	51 (71.8)	19 (59.4)	
70–79	13 (40.6)	19 (59.4)	
Level of education			
No formal education	151 (81.6)	34 (18.4)	0.01*
Primary	63 (90.0)	7 (10.0)	
≥Secondary	84 (93.3)	6 (6.7)	
Caste/ethnicity			
Upper cast	117 (88.0)	16 (12.0)	0.46
Janajati	163 (85.3)	28 (14.7)	
Dalit	18 (85.7)	3 (14.3)	
Marital status			
Married	277 (87.9)	38 (12.1)	0.006*
Widow or widower	21 (70.0)	9 (30.0)	
Occupation^a^			
Employ	36 (90.0)	4 (10.0)	0.001*
Self employed	77 (98.7)	1 (1.3)	
Unemployed	168 (84.0)	32 (16.0)	
Retired	17 (63.0)	10 (37.0)	
Economic status			
Poor (≤NRs 8498)	271 (86.3)	43 (13.7)	0.9
Higher (>NRs 8498)	27 (87.1)	4 (12.9)	

Chi square test was done

* Is significant

^a^Fischer exact test

### Socio-demographic factors associated with elevated 10-year CVD risk

CVD risk was significantly (p = 0.01) associated with the level of education. Of participants without formal education, 18.4% had elevated CVD risk. However, it was 10% and 6.7% of participants who had a primary and secondary level of education. Elevated CVD risk was higher among Janajati and Dalit (14.7% and 14.3%) in comparison to upper caste participants (12%). Participants whose partner was not alive (30%) had higher CVD risk compared to married (12.1%). Occupation was significantly (p = 0.001) associated with CVD risk. Of students, homemaker or non-paid workers, 16% had elevated CVD risk. Similarly, among retired participants, 37% had more than 10% CVD risk. However, the elevated risk was only 1.3% and 10% among self-employed and employed participants, respectively (Table 3).

### Proportion of population who need immediate drug therapy

The estimated proportion of population who need drug therapy was 8.6% using threshold of >30% risk and while it was 11% at threshold level of >20% risk. However, such estimated proportion was more than threefold (36%) using single risk factor approach. Proportion of men who required drug intervention was 11.7% and 14.3% at threshold of >30% and >20% CVD risk, respectively. While proportion of women was 6.3% and 8.4% at same threshold level (Table 4).

**Table 4:** Study population requiring pharmacotherapy using WHO/ISH chart at different threshold levels and single risk factors approach, n (%)

Approach	Variants	Persons requiring drugs					
		Threshold CV risk >30%			Threshold CV risk >20%		
		Male	Female	Total	Male	Female	Total
WHO/ISH chart	Chart	2 (1.3)	4 (2.0)	6 (1.8)	7 (4.5)	8 (4.2)	15 (4.3)
	Chart + BP^a^	16 (10.6)	8 (4.2)	24 (6.9)	15 (9.7)	8 (4.2)	23 (6.6)
	Total	18 (11.7)	12 (6.3)	30 (8.6)^b^	22 (14.3)	16 (8.4)	38 (11)^c^
Single risk factor	BP ≥ 140/90	58 (38.7)	52 (27.2)	110 (31.9)	58 (38.7)	52 (27.2)	110 (31.9)
	TC ≥ 6	8 (5.2)	6 (3.1)	14 (4.1)	8 (5.2)	6 (3.1)	14 (4.1)
	Total	64 (43.9)	58 (30.3)	124 (36)	64 (43.9)	58 (30.3)	124 (36)

^a^BP ≥ 160/100 mmHg

^b^Chart with BP ≥ 160/100 mmHg vs single risk factor, at >30% threshold, χ^2^ = 64, p = 0.001

^c^Chart with BP ≥ 160/100 mmHg vs single risk factor, at >20% threshold, χ^2^ = 64.5, p = 0.001

## Discussion

Our study had an objective to estimate total 10-year CVD risk among study population. This study found that large proportion of study participants had moderate to high risk (13.6%) which was consistent with the findings reported in national and international studies conducted in similar settings. This proportion of population at elevated risk of CVD was almost same in one sample of Nepal (13.4%) reported by Mendis et al. [7], while higher than the finding of another study conducted in a sub-urban population of Nepal (9.5%) [20]. However, the proportion of raised risk in current study was slightly lower than that of Kathmandu (14.6%) [26] and rural India (16%) [27]. Regarding the proportion of population under high risk of CVD, it was slightly higher in our study (4.3%) than that of study conducted in Malaysia (2.3%) and Cambodia (1.3%) and lower than that of Mangolian study (6%) [12]. Similar heterogeneous results were observed in different countries (China 1.1%, Iran 1.7%, Sri Lanka 2.2%, Cuba 2.8%, Nigeria 5.0%, Georgia 9.6%, Pakistan 10.0%) [7]. Very high-risk (>30%) was 1.7% in our study, which was slightly lower than the estimated for age 40–65 years throughout the country of Nepal (3.2%) [28]. These different data revealed that the population under risk for development of stroke and coronary heart disease were high even in a rural community of Nepal. Application of WHO/ISH chart helps to categorize population into different risk levels. Population-based lifestyle modification strategy can be applied to low-risk population while individual counseling and frequent follow-up assessment need to be added for moderate risk population. However, more rigorous treatment strategies would be needed for the population at high and very high-risk category [16].

CVD risk was significantly associated with the level of education. A study conducted in Vienna echoed the same finding that education is associated with the level of understanding on risk factors [29]. Evidence also suggests that increasing knowledge on risk factors can substantially motivate people for changing risk behaviors [30]. This fact suggests that we need to improve awareness and knowledge about prevention of cardiovascular diseases. Occupation was significantly associated with elevated CVD risk. A higher proportion of unemployed and retired participants had raised risk. One study of Mauritius also proved the association of occupation and cardiovascular risk, mostly related to physical activities and economic status [31]. These findings provide the evidence that we should target unemployed population for early prevention of cardiovascular disease. As expected, age-related increase in CVD risk was associated with an increase in risk factor level [32].

As explained in the WHO guideline for assessment and management of cardiovascular disease [16], individuals who have blood pressure of ≥160/100 mm Hg need appropriate drug therapy even though they are in low and moderate CVD risk category. However, there is still no universal consensus for appropriate threshold (30% vs 20%) of total CVD risk at which drug treatment are initiated [7, 16]. It depends on the availability of resource of a country and impact of the specific intervention. Current data reveals that we can prevent large numbers of ischemic heart disease and stroke incidence if threshold is reduced from >30 to >20% risk. Our result demonstrated that about 11% would be treated by drug if >20% risk was taken as threshold while about 8.6% would need drug at threshold of >30% risk. However, about threefold more individual would require pharmacotherapy if we target single risk factors like hypertension and raised cholesterol. Our estimation of proportion of the population in need of drug therapy was slightly lower than that of Bangladesh at same threshold level (8.3% and 4%). However, as in our study, population who require drug were almost threefold higher by targeting single risk factors (28.2%) [33]. Our estimation was comparable to another study conducted at Cuba [34], where sample population requiring drug at aforementioned threshold level were 12.5% and 11.5% with total risk approach restricting about threefold of the population from over prescription of drug than single risk factor approach (43.8%). Similar result was found in a study in Africa [7, 9]. Therefore, high proportion of the population were in need of pharmacotherapy and it would prevent over prescription of the drug if total CVD risk approach applied to prevent CVD.

Due to lack of data related to cardiovascular morbidity and mortality, WHO/ISH charts have not validated yet in Nepal. The chart might have underestimated the total CVD risk, as it did not include the individual with antihypertensive therapy, raised triglyceride, low HDL, family history of CVD and obesity. Higher number of janajati participants were involved in the study. Therefore, findings of the study should cautiously be generalized to other rural population of Nepal.

## Conclusion

A large proportion of Nepalese rural population is at moderate and high CVD risk. Immediate pharmacological interventions are warranted for at least one in every ten individuals along with lifestyle interventions. This situation underlines the importance of immediate designing and implementation of both population-wise and high-risk approaches to minimize CVD burden in the future. Total risk approach would be more effective for initiation of preventive pharmacotherapy compared to the single risk factor approach to allocate the limited resources more efficiently.

## Abbreviations

CVD: Cardiovascular Diseases
LMIC: Low and Middle Income Countries
NCD: Noncommunicable Diseases
WHO/ISH: World Health Organization/International Society for Hypertension
STEPS: Stepwise Approach to Surveillance
SEAR: South East Asia Region

## References

[1] World Health Organization (2014). Global status report on noncommunicable diseases 2014.

[2] Mathers CD, Loncar D (2006). Projections of global mortality and burden of disease from 2002 to 2030. PLoS Med.

[3] World Health Organization (2011). Non communicable diseases in the South East Asia region: situation and response 2011.

[4] Bhandari G, Angdembe M, Dhimal M, Neupane S, Bhusal C (2014). State of non-communicable diseases in Nepal. BMC Public Health.

[5] Emberson J, Whincup P, Morris R, Walker M, Ebrahim S (2004). Evaluating the impact of population and high-risk strategies for the primary prevention of cardiovascular disease. Eur Heart J.

[6] Hobbs FDR (2004). Cardiovascular disease: different strategies for primary and secondary prevention?. Heart.

[7] Mendis S, Lindholm LH, Anderson SG, Alwan A, Koju R, Onwubere BJ, Kayani AM, Abeysinghe N, Duneas A, Tabagari S (2011). Total cardiovascular risk approach to improve efficiency of cardiovascular prevention in resource constrain settings. J Clin Epidemiol.

[8] Batsis J, Lopez-Jimenez F (2010). Cardiovascular risk assessment—from individual risk prediction to estimation of global risk and change in risk in the population. BMC Med.

[9] Ndindjock R, Gedeon J, Mendis S, Paccaud F, Bovet P (2011). Potential impact of single-risk-factor versus total risk management for the prevention of cardiovascular events in Seychelles. Bull World Health Organ.

[10] Wilson PWF, D’Agostino RB, Levy D, Belanger AM, Silbershatz H, Kannel WB (1998). Prediction of coronary heart disease using risk factor categories. Circulation.

[11] Kannel WB, McGee D, Gordon T (1976). A general cardiovascular risk profile: the Framingham Study. Am J Cardiol.

[12] D’Agostino RB, Vasan RS, Pencina MJ, Wolf PA, Cobain M, Massaro JM, Kannel WB (2008). General cardiovascular risk profile for use in primary care: the Framingham Heart Study. Circulation.

[13] Conroy RM, Pyorala K, Fitzgerald AP, Sans S, Menotti A, De Backer G, De Bacquer D, Ducimetiere P, Jousilahti P, Keil U (2003). Estimation of ten-year risk of fatal cardiovascular disease in Europe: the SCORE project. Eur Heart J.

[14] Julia H-C, Carol C, John R, Peter B (2010). Derivation, validation, and evaluation of a new QRISK model to estimate lifetime risk of cardiovascular disease: cohort study using QResearch database. BMJ.

[15] Woodward M, Brindle P, Tunstall-Pedoe H (2007). Adding social deprivation and family history to cardiovascular risk assessment: the ASSIGN score from the Scottish Heart Health Extended Cohort (SHHEC). Heart.

[16] World Health Organization (2007). Prevention of cardiovascular disease: Guideline for assessment and management of cardiovascular risk.

[17] Mendis S, Lindholm LH, Mancia G, Whitworth J, Alderman M, Lim S, Heagerty T (2007). World Health Organization (WHO) and International Society of Hypertension (ISH) risk prediction charts: assessment of cardiovascular risk for prevention and control of cardiovascular disease in low and middle-income countries. J Hypertens.

[18] Otgontuya D, Oum S, Buckley B, Bonita R (2013). Assessment of total cardiovascular risk using WHO/ISH risk prediction charts in three low and middle income countries in Asia. BMC Public Health.

[19] Central Bureau of Statistics (2014). National Population and Housing Census 2011, vol. 05, 1st edn.

[20] Koju R, Gurung R, Pant P, Humagain S, Yogol CM, Koju A, Manandhar K, Karmacharya B, Bedi TRS (2011). Prediction of cardiovascular disease in suburban population of 3 municipalities in Nepal. Nepal Heart J.

[21] World Health Organization (2005). WHO STEPS surveillance manual: the WHO STEPwise approach to chronic disease risk factor surveillance/noncommunicable diseases and mental health.

[22] WHO/ISH risk prediction charts for 14 WHO epidemiological sub-regions. http://ish-world.com/downloads/activities/colour_charts_24_Aug_07.pdf.

[23] World Health Organization (2007). Prevention of cardiovascular diseases: pocket guidelines for assessment and management of cardiovascular risk.

[24] World Health Organization (2006). Definition and diagnosis of diabetes mellitus and intermediate hyperglycemia: report of a WHO/IDF consultation.

[25] Central Bureau of Statistics (2011). Nepal living standards survey 2010/11.

[26] Dhungana RR, Khanal MK, Pandey AR, Thapa P, Devkota S, Mumu SJ, Shayami A, Ali L (2015). Assessment of short term cardiovascular risk among 40 years and above population in a selected community of Kathmandu, Nepal. J Nepal Health Res Counc.

[27] Ghorpade AG, Shrivastava SR, Kar SSSS, Majgi SM, Roy G (2015). Estimation of the cardiovascular risk using World Health Organization/International Society of Hypertension (WHO/ISH) risk prediction charts in a rural population of South India. Int J Health Policy Manag.

[28] Aryal KN, Mehata S, Vaidya A, Singh S, Paulin F, Madanlal RG, Riley LM, Cowan M, Guthold R, Singh SP, Bhusal CL, Lohani GR. Non communicable diseases risk factors: STEPS Survey Nepal 2013. Kathmandu; 2014.

[29] Samal D, Greisenegger S, Auff E, Lang W, Lalouschek W (2007). The relation between knowledge about hypertension and education in hospitalized patients with stroke in Vienna. Stroke.

[30] McDermott MM, Mandapat AL, Moates A, Albay M, Chiou E, Celic L, Greenland P (2003). Knowledge and attitudes regarding cardiovascular disease risk and prevention in patients with coronary or peripheral arterial disease. Arch Intern Med.

[31] Pereira MA, Kriska AM, Collins VR, Dowse GK, Tuomilehto J, Alberti KG, Gareeboo H, Hemraj F, Purran A, Fareed D (1998). Occupational status and cardiovascular disease risk factors in the rapidly developing, high-risk population of Mauritius. Am J Epidemiol.

[32] Jousilahti P, Vartiainen E, Tuomilehto J, Puska P (1999). Sex, age, cardiovascular risk factors, and coronary heart disease: a prospective follow-up study of 14,786 middle-aged men and women in Finland. Circulation.

[33] Fatema K, Zwar NA, Milton AH, Rahman B, Ali L (2015). Application of two versions of the WHO/international society of hypertension absolute cardiovascular risk assessment tools in a rural Bangladeshi population. BMJ Open.

[34] Nordet P, Mendis S, Duenas A, de la Noval R, Armas N, de la Noval IL, Pupo H (2013). Total cardiovascular risk assessment and management using two prediction tools, with and without blood cholesterol. MEDICC Rev.

